# Differentiation/Purification Protocol for Retinal Pigment Epithelium from Mouse Induced Pluripotent Stem Cells as a Research Tool

**DOI:** 10.1371/journal.pone.0158282

**Published:** 2016-07-06

**Authors:** Yuko Iwasaki, Sunao Sugita, Michiko Mandai, Shigenobu Yonemura, Akishi Onishi, Shin-ichiro Ito, Manabu Mochizuki, Kyoko Ohno-Matsui, Masayo Takahashi

**Affiliations:** 1 Laboratory for Retinal Regeneration, RIKEN Center for Developmental Biology, Kobe, Hyogo, Japan; 2 Department of Ophthalmology & Visual Science, Graduate School of Medical and Dental Science, Tokyo Medical and Dental University, Bunkyo-ku, Tokyo, Japan; 3 Ultrastructural Research Team, RIKEN Center for Life Science Technologies, Kobe, Hyogo, Japan; 4 Department of Cell Biology, Institute of Biomedical Sciences, Tokushima University Graduate School, Tokushima, Japan; University of Newcastle upon Tyne, UNITED KINGDOM

## Abstract

**Purpose:**

To establish a novel protocol for differentiation of retinal pigment epithelium (RPE) with high purity from mouse induced pluripotent stem cells (iPSC).

**Methods:**

Retinal progenitor cells were differentiated from mouse iPSC, and RPE differentiation was then enhanced by activation of the Wnt signaling pathway, inhibition of the fibroblast growth factor signaling pathway, and inhibition of the Rho-associated, coiled-coil containing protein kinase signaling pathway. Expanded pigmented cells were purified by plate adhesion after Accutase^®^ treatment. Enriched cells were cultured until they developed a cobblestone appearance with cuboidal shape. The characteristics of iPS-RPE were confirmed by gene expression, immunocytochemistry, and electron microscopy. Functions and immunologic features of the iPS-RPE were also evaluated.

**Results:**

We obtained iPS-RPE at high purity (approximately 98%). The iPS-RPE showed apical-basal polarity and cellular structure characteristic of RPE. Expression levels of several RPE markers were lower than those of freshly isolated mouse RPE but comparable to those of primary cultured RPE. The iPS-RPE could form tight junctions, phagocytose photoreceptor outer segments, express immune antigens, and suppress lymphocyte proliferation.

**Conclusion:**

We successfully developed a differentiation/purification protocol to obtain mouse iPS-RPE. The mouse iPS-RPE can serve as an attractive tool for functional and morphological studies of RPE.

## Introduction

Regenerative therapy using differentiated cells derived from stem cells is drawing attention worldwide. We have been conducting a clinical study on the autologous transplantation of retinal pigment epithelium (RPE) derived from induced pluripotent stem cells (iPSC) in a patient with age-related macular degeneration. Human iPS-RPE have been evaluated for safety (eg, tumorigenesis), the ability to support photoreceptor cells, and the ability to suppress lymphocyte reactions in rat and mouse models [1–4]. Although clinical trials of iPSC or embryonic stem cells (ESC, [5]) are already on the way, it is important to know how transplanted differentiated RPE would survive and retain proper functions in diseased eyes.

The engraftment process of iPS-RPE is composed of various intercellular communications. The immune system condition of the recipient, the capacity of iPS-RPE to survive in inflammatory intraocular conditions, and the ability of iPS-RPE to attach to the diseased extracellular matrix and to make lateral connections between diseased RPE of recipients are important for cell survival and function. Additionally, the immunologic features of graft RPE are also important, because RPE suppresses pro-inflammatory lymphocytes [1, 6, 7]. Understanding these mechanisms is important for gaining beneficial effects from transplantation, including contributing to the patients’ quality of vision, and standardizing regenerative medicine techniques. For these purposes, in vivo experiments using animal models are essential, and mouse RPE cells are still in high demand because there are various types of eye disease model mice that are suitable as recipients and there are also various types of genetically labeled or modified mice useful for detailed studies.

Mouse primary RPE (pRPE) has been widely used as a research tool for understanding the various characteristics of RPE [6, 8–13]. Some researchers obtained pRPE from postnatal mice, and others obtained pRPE from adult mice. The obtained pRPE was sometimes used immediately after isolation and sometimes used after several days to weeks of culture, with or without passages or immortalization. Each method was selected by each researcher according to the purpose of their studies. It is difficult to obtain a substantial number of pRPE cells without loss of the cuboidal shape.

Cell-to-cell contact depends on the quality and quantity of cell adhesion molecules, which are expressed on the cell surface [14, 15]. Hence, as a research tool for understanding the engraftment process of human iPS-RPE, the cell morphology should be similar to that of human iPS-RPE, which exhibits the cuboidal morphology of RPE [2]. It is essential to be able to consistently obtain a substantial number of cells for research purposes. If we could obtain RPE differentiated from mouse iPSC or ESC in a substantial quality and quantity, such RPE would be an attractive tool for understanding the in vivo process that occurs after human iPS-RPE transplantation.

Many investigators reported that RPE can be differentiated and purified from human iPSC and ESC [2, 16, 17]. Several reports showed RPE could also be differentiated from mouse iPSC and ESC in part of the ocular structure [18, 19]. However, as far as we know, there are no previous reports that describe the protocol for differentiation of purified mouse iPS-RPE. In the present study, we describe a protocol for differentiation of mouse iPS-RPE with high purity and evaluate the characteristics of these cells. We also provided detailed conditions of trial and error to share our process in optimizing the subsequent protocol.

The protocol is divided into four parts: (1) induction of retinal progenitor cells, (2) adherence to laminin-coated dishes and fate induction to RPE, (3) purification of pigmented cells, and (4) further culture until cells develop a cuboidal shape. We then compared characteristics of iPS-RPE with RPEs of primary cultures and also evaluated cellular functions including immunologic features.

## Results

### Generation of pigment cells from retinal progenitor cells derived from mouse induced pluripotent stem cells (iPSC)

**Fig 1:**
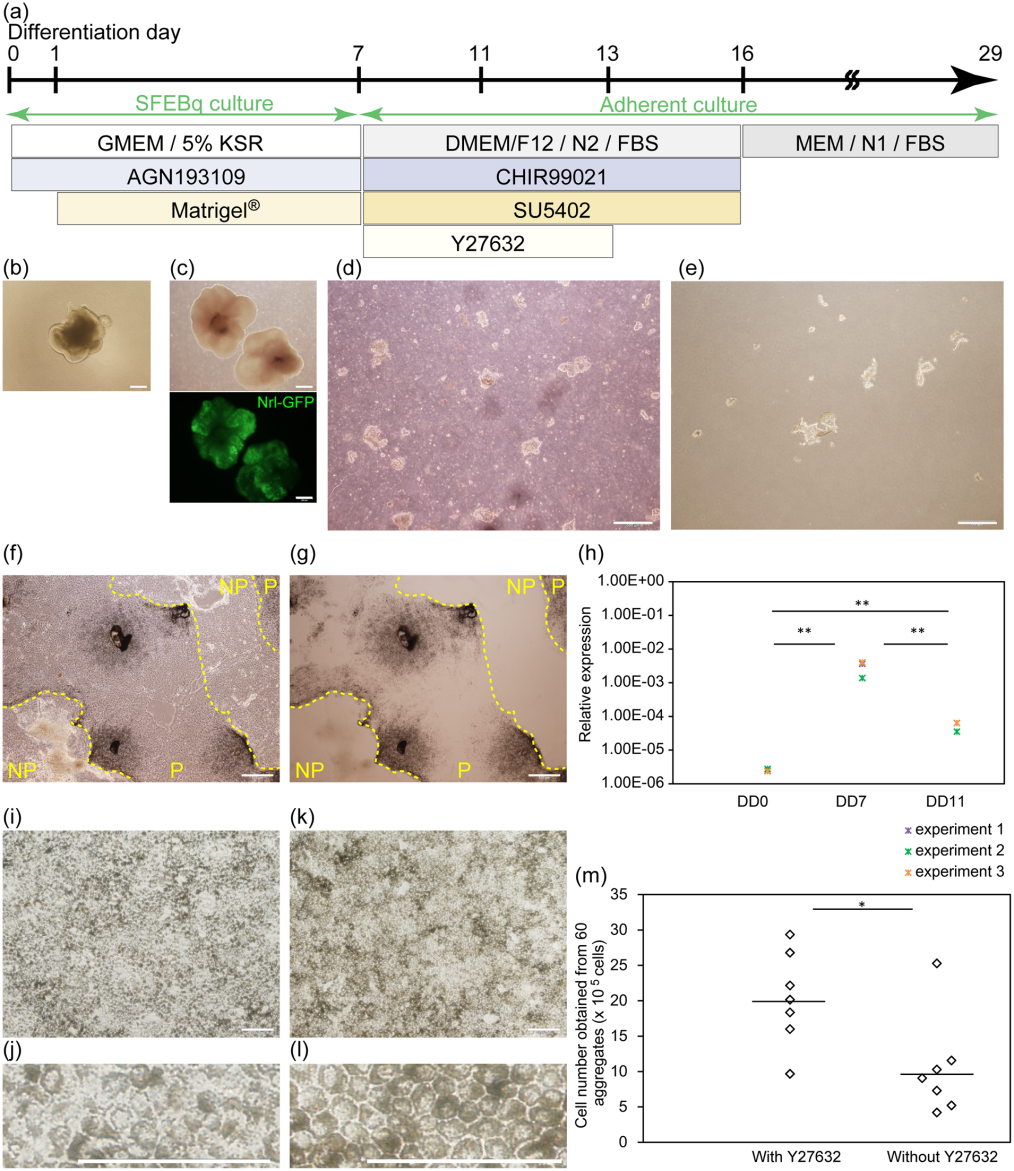
Differentiation and purification of pigmented cells from Nrl-GFP mouse iPSC. (a) Schematic of the procedure. SFEBq; serum-free floating culture of embryoid body-like aggregates with quick reaggregation, KSR; knockout serum replacement. (b) Appearance of aggregate at differentiation day (DD) 7. (c) Differentiation into retinal structure was confirmed by evaluation of Nrl-GFP at DD26. Optical and fluorescence images are shown. (d) Broken up aggregates. (e) Large cell clumps and sheets were collected. (f and g) Phase-contrast (f) and bright field (g) images of pigmented (P) and non-pigmented (NP) colonies at DD11 are shown. (h) Time-dependent change of relative expression of Rx evaluated by qRT-PCR. Data from three independent experiments are shown in different colors. Each mark indicates the mean value of triplicate evaluations. Tukey-Kramer test. n = 3. (i-l) Phase-contrast images at DD16 (i and j) and at DD29 (k and l) are shown. Magnified images (j and l) show the cobblestone appearance at DD29. (m) Pigmented cell number obtained from 60 aggregates with or without Y27632. Mann-Whitney U test. Data from seven independent experiments are shown. Bar indicates the median values. Scale bars, 500 μm (c-g), 200 μm (b) and 100 μm (i-l). **: p<0.01, *: p<0.05.

The outline of our RPE differentiation protocol is shown in Fig 1a. At differentiation day (DD) 7, a rigid epithelial structure is formed on the surfaces of aggregates, as previously reported (Fig 1b, [19]). The ability of DD7 aggregates to differentiate into retinal structures was confirmed by evaluating the Nrl-GFP expression at DD26 (Fig 1c). DD7 aggregates were broken up into large cell clumps or sheets mechanically by needles (Fig 1d) and were collected by centrifugation. Cell fragments and debris were removed by filtration. The remaining cell clumps and sheets (Fig 1e) were placed on laminin-coated dishes in retinal maturation medium 2 supplemented with CHIR99021 (inhibitor of glycogen synthase kinase-3β, activator of Wnt signaling pathway) and SU5402 (inhibitor of fibroblast growth factor receptor and vascular endothelial growth factor receptor), which are both important factors for the fate decision between retina and RPE [19–22]. Y27632 (inhibitor of Rho-associated, coiled-coil containing protein kinase) was also added to the medium.

Seeded cell clumps and sheets attached and expanded concentrically by DD11. Some colonies became pigmented, and others did not (Fig 1f and 1g; P indicates pigmented colonies, and NP indicates non-pigmented colonies). Expression of Rx, a marker of retinal progenitor cells [23], increased between DD0 and DD7 and decreased between DD7 and DD11 (Fig 1h). At DD11, non-pigmented colonies and less adhesive pigmented cells were removed by a 5-min treatment with Accutase^®^ and pipetting. Small amounts of adhesive non-pigmented cells were removed manually (S1a and S1b Fig). The remaining adhesive pigmented cells were dissociated by 3 min of treatment with 0.25% trypsin-1 mM EDTA and were passaged in laminin111-coated wells. Passaged cells proliferated and became confluent by DD16 (Fig 1i and 1j), and the medium was changed to MEM / N1 / FBS medium. By DD29, cell alignment improved and the cells developed a cobblestone appearance with cuboidal shape, which is a characteristic feature of retinal pigment epithelium (RPE, Fig 1k and 1l).

When large cell clumps and cell sheets were dissociated into single cells and plated without Y27632 at DD7, the cell attachment was poor and pigmentation did not occur. However, cell attachment improved when the medium was supplemented with Y27632, or the cells were attached as a sheet structure (S1c Fig). Furthermore, when cells were seeded as a sheet structure with Y27632, we could obtain a significantly larger number of pigmented cells at DD11 (with Y27632: median 2.02×10^6^ cells per 60 aggregates with a range of 9.68×10^5^ to 2.94×10^6^ cells, without Y27632: median 9.07×10^5^ cells per 60 aggregates with a range of 4.21×10^5^ to 2.53×10^6^ cells, from seven independent experiments, p<0.05, Mann-Whitney U test, Fig 1m). Y27632 was also effective for the attachment of cells at the passage on D11 (S1d Fig).

As to coating materials that we used at the DD11 passage, we tried various materials before deciding on laminin111, such as fibronectin, collagen type 4, gelatin, collagen type 1, ECM^®^, CELLstart^®^, and Matrigel^®^. The cells could not form sheet structures on collagen type1 and Matrigel^®^ (40% and 100%, S1e Fig); however, the cells could form a sheet structure on the other coating materials. From our observations, cell attachment and formation of sheet structures were rather influenced by the dish material. When iPS-RPE was maintained on slide glass, patchy regions with loss of ZO-1 expression were sometimes seen (8 wells in 28 wells on slide glass, 0 wells in 58 wells on a 96-well plate, both from five independent experiments, p<0.01, chi-square test, S1f–S1h Fig).

### Differentiated pigmented cells were polarized epithelial cells

**Fig 2:**
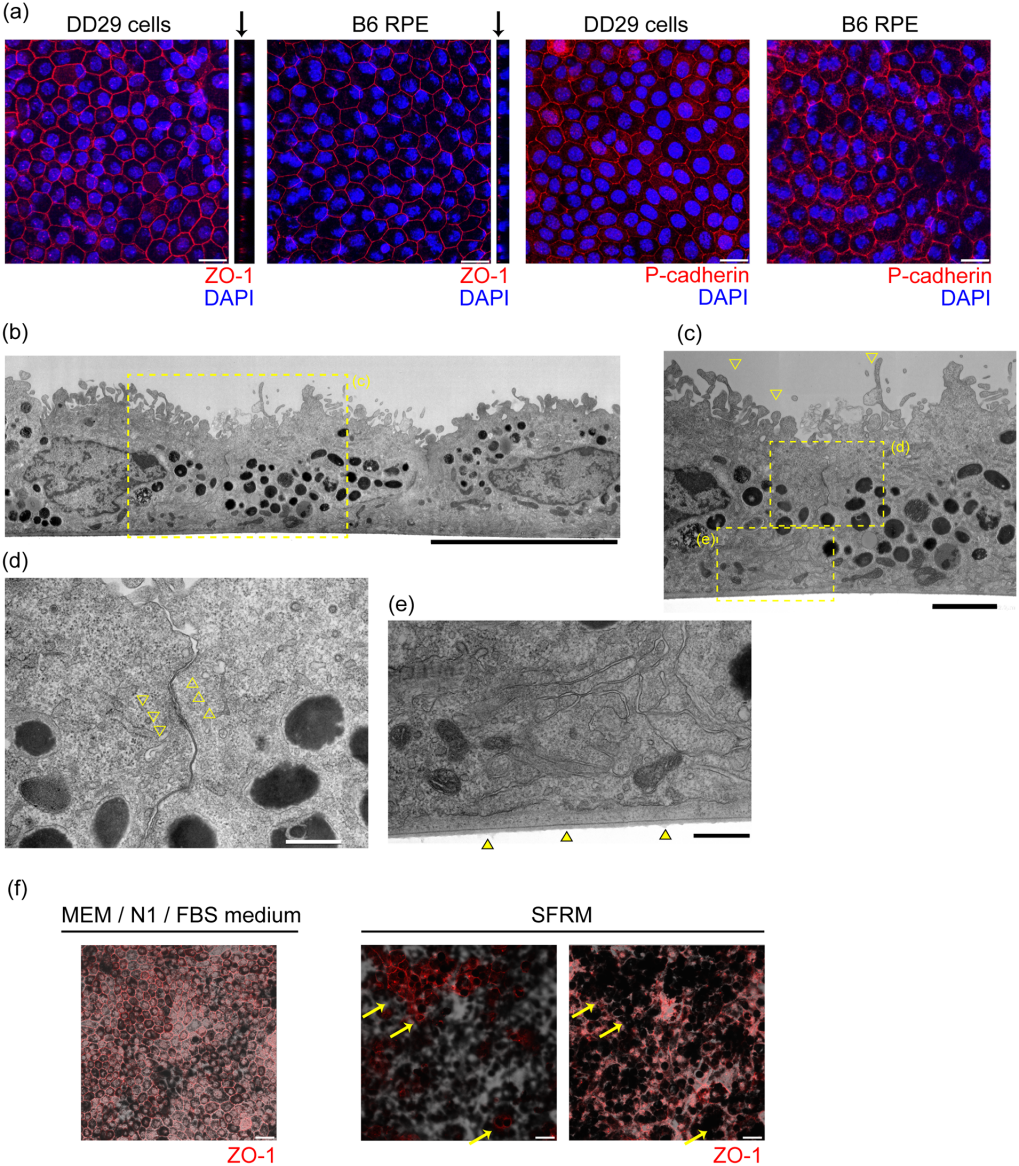
Morphological evaluation of DD29 cells. (a) Confocal imaging of ZO-1 and P-cadherin in the DD29 cells and PN day10-mouse RPE. The polarized expression pattern of ZO-1 on the apical side of the cells is shown (black arrows). Nuclei were counter-stained with DAPI. (b) Electron microscopy image of the DD29 cells. Magnified views of the areas of the yellow dotted squares show the presence of microvilli (c), intercellular attachment (d), and extracellular matrix (e). (f) Immunocytochemistry of ZO-1 of DD29 cells maintained by MEM / N1 / FBS medium and serum-free RPE medium (SFRM). Most cells maintained by MEM / N1 / FBS medium were in a cuboidal shape and formed a monolayer structure. ZO-1-positive cell clumps (arrows) in a different plane of Z-scan imaging with increased cell sizes was observed when cells were maintained with SFRM. Scale bars: 30um (f), 20 μm (a), 10 μm (b), 2 μm (c) and 0.5 μm (d and e).

Next, we evaluated whether differentiated pigmented cells showed the morphology that is characteristic of RPE by using immunocytochemistry (ICC, Fig 2a). Most cells had a hexagonal shape. The cells expressed ZO-1 and P-cadherin. ZO-1 was localized on the apical side in the junction between adjacent cells (arrows on Z-stack images), which is the same orientation as in the RPE of postnatal (PN) day 10-mice. Polarity of the apical-basal axis was further confirmed by electron microscopy (Fig 2b–2e). The pigmented cells formed microvilli in the apical sides (Fig 2c). Dense intercellular attachment was observed (Fig 2d), and extracellular matrix was present on the basal side (Fig 2e). Most cells were in a cuboidal shape and formed a monolayer structure as implied by the relatively uniform staining pattern of ZO-1 on a single plane of Z-stack imaging (Fig 2f). ZO-1-positive cell clumps (arrows) in a different plane of Z-scan imaging with increased cell sizes was observed when cells were maintained with serum-free RPE medium (SFRM) supplemented with basic fibroblast growth factor and SB431542, which we use for human iPS-RPE [2]. The nuclear staining pattern of Sox9, which is an RPE marker [24], also was confirmed by immunocytochemistry in the monolayer pigmented cells that were maintained with MEM / N1 / FBS medium (S2a Fig).

Pigmented cells that were not passaged on DD11 exhibited fine, long microvilli at DD29 (S2b and S2c Fig). However, cells in the peripheral region did not become pigmented, and cuboidal morphology was not observed (S2d and S2e Fig). By passaging at DD11, cells consistently proliferated to confluency, and most cells acquired cuboidal morphology (S2f Fig).

Based on these morphological features evaluated by ICC together with electron microscopy, and confirmation that DD7 aggregates have the ability to differentiate into retinal cells, we defined these pigmented cells derived from our protocol as iPS-RPE.

### Purity and characteristics of iPS-RPE

**Fig 3:**
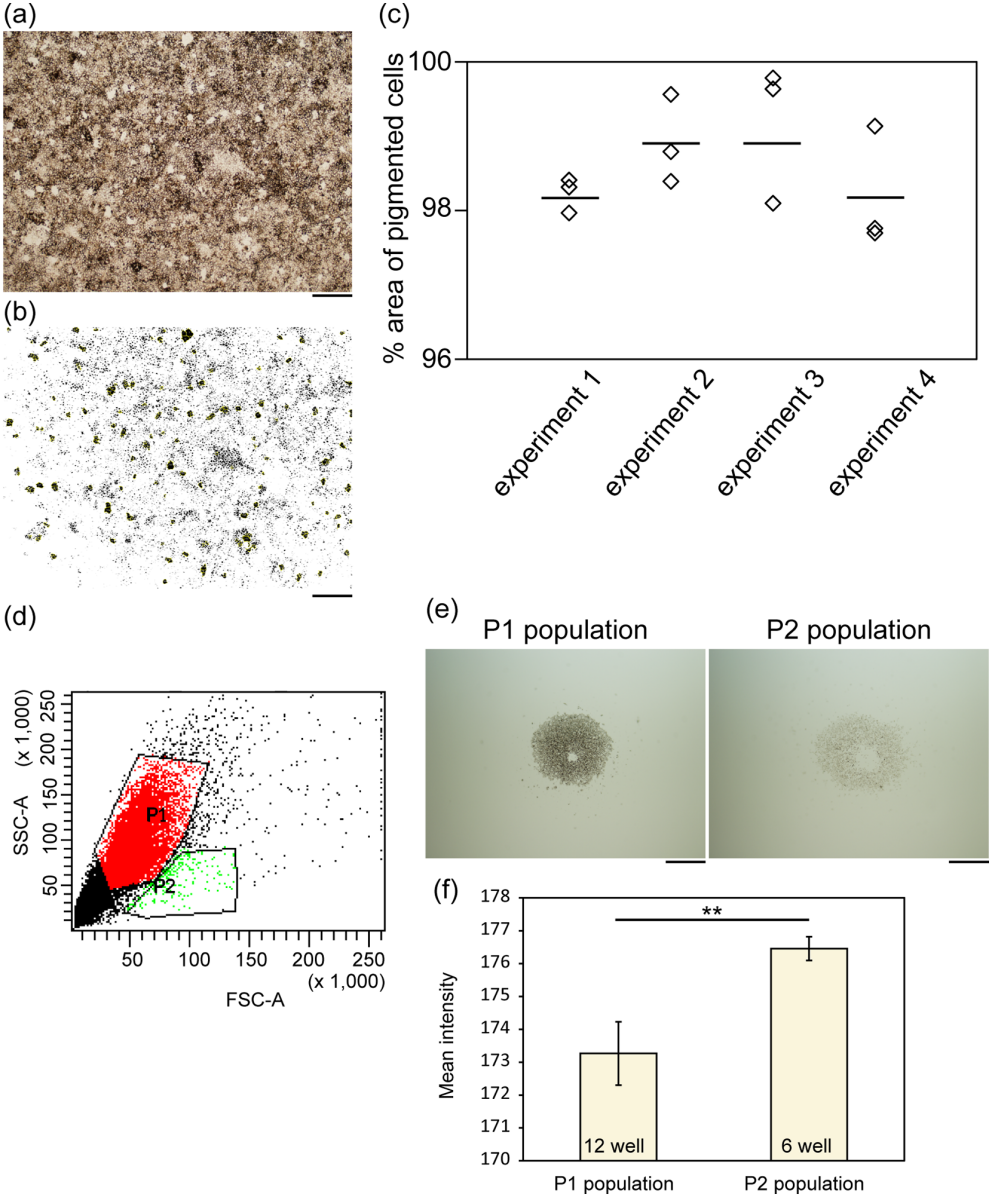
Purity of iPS-RPE. (a and b) Representative photograph of DD29 iPS-RPE. Bright-field photos (a) were analyzed by Image J software to obtain the number of pixels representing the area of less pigmented cells, as shown in the black region surrounded by a yellow line in (b). (c) Proportion of pigmented cells to total area of the images was expressed as a percent. Three wells were evaluated in each experiment, and data from four independent experiments are shown. Bars indicate the mean value. (d) Representative data of flow cytometry analysis evaluated by side-scattering (SSC) parameter and forward-scattering (FSC) parameter. There was a main population (P1) in addition to a subpopulation with relatively low SSC (P2). (e) Bright-field photos of 5000 cells/well from the P1 and P2 populations. (f) Pigmentation of the P1 population was more intense than that of the P2 population. Mann-Whitney U test. n = 12 for ‘P1 population’ group, n = 6 for ‘P2 population’ group. Scale bars: 500 μm (e), 200 μm (a, b). **: p<0.01. Error bars in (f) represent S.D.

We evaluated the purity of iPS-RPE by image analysis of bright field photos. Purity of iPS-RPE was 98.6% ± 0.74% (mean ± S.D. of 12 wells from four independent experiments, Fig 3a–3c). Furthermore, when we analyzed the iPS-RPE by flow cytometry, we could recognize the main population with relatively high side-scattering (SSC) parameter (P1) and subpopulation with relatively low SSC parameter (P2, Fig 3d). Pigmentation of the P1 population was more intense than that of the P2 population (p<0.01, Mann-Whitney U test, Fig 3e and 3f).

**Fig 4:**
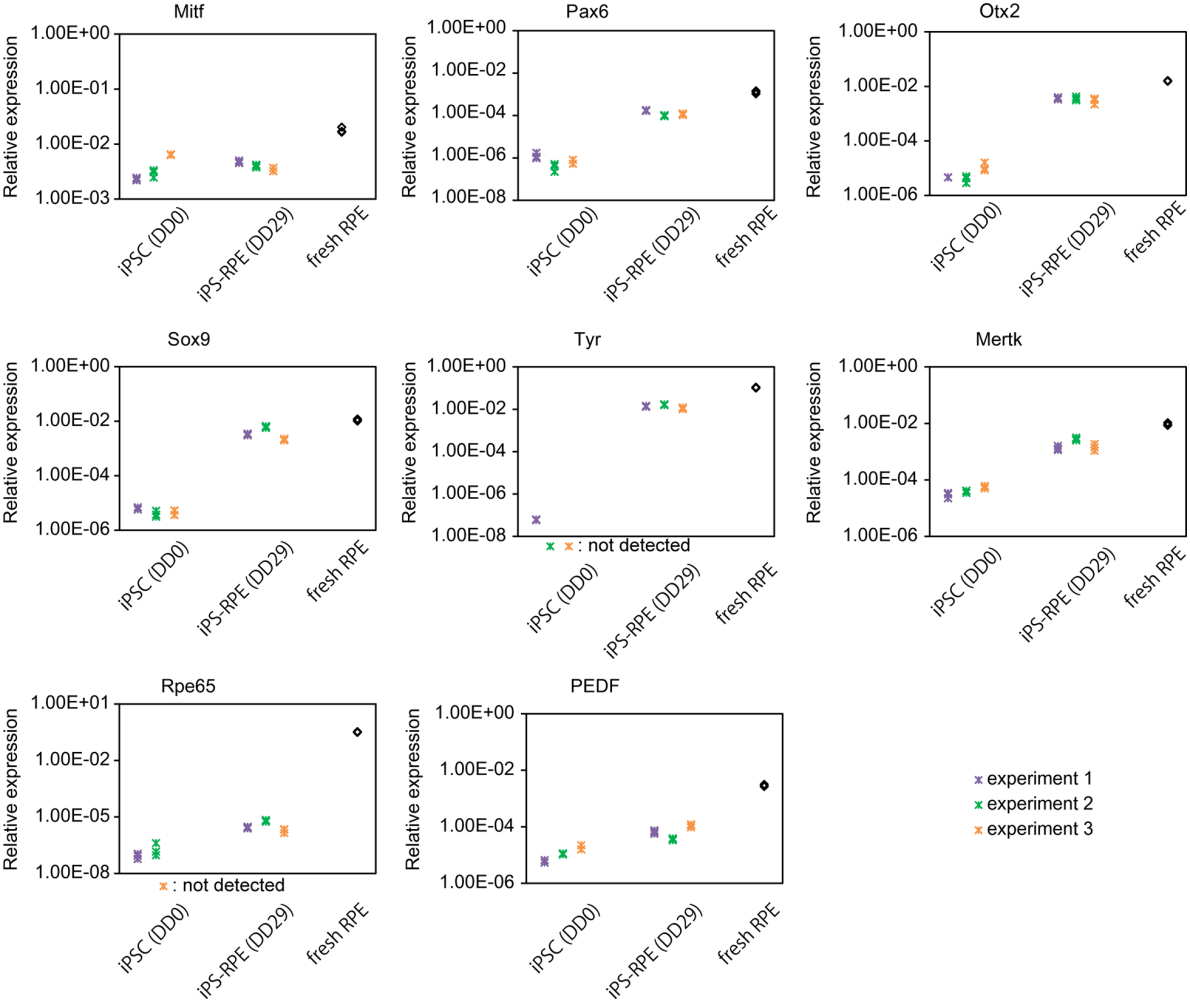
Expression of developmental and functional markers of RPE. Transcriptional analysis of iPSC (DD0), iPS-RPE (DD29) and control RPE freshly obtained from PN day10-mice is shown. Values were normalized by Gapdh. The expression levels of each marker are presented as triplicate data from three independent experiments with different colors.

The expression of RPE developmental marker mRNAs in iPSC (DD0), iPS-RPE (DD29) and control RPE freshly obtained from PN day10-mice is shown in Fig 4. Mitf and Pax6, which are expressed during development of RPE and decrease with maturation [25–27], were expressed in iPS-RPE at lower levels than in RPE freshly obtained from PN day 10-mice. Otx2 and Sox9, which are expressed from the middle phase of RPE development and continue to be expressed during the adulthood [24, 28], were highly expressed in iPS-RPE, but the expression levels were slightly lower than those in PN day10-RPE. RPE functional genes Tyr, Mertk, Serpinf1 (PEDF), and Rpe65 were expressed at higher levels in iPS-RPE than in iPSC, but at lower levels than in PN day10-RPE.

### Functional characteristics of mouse iPS-RPE as a research tool

**Fig 5:**
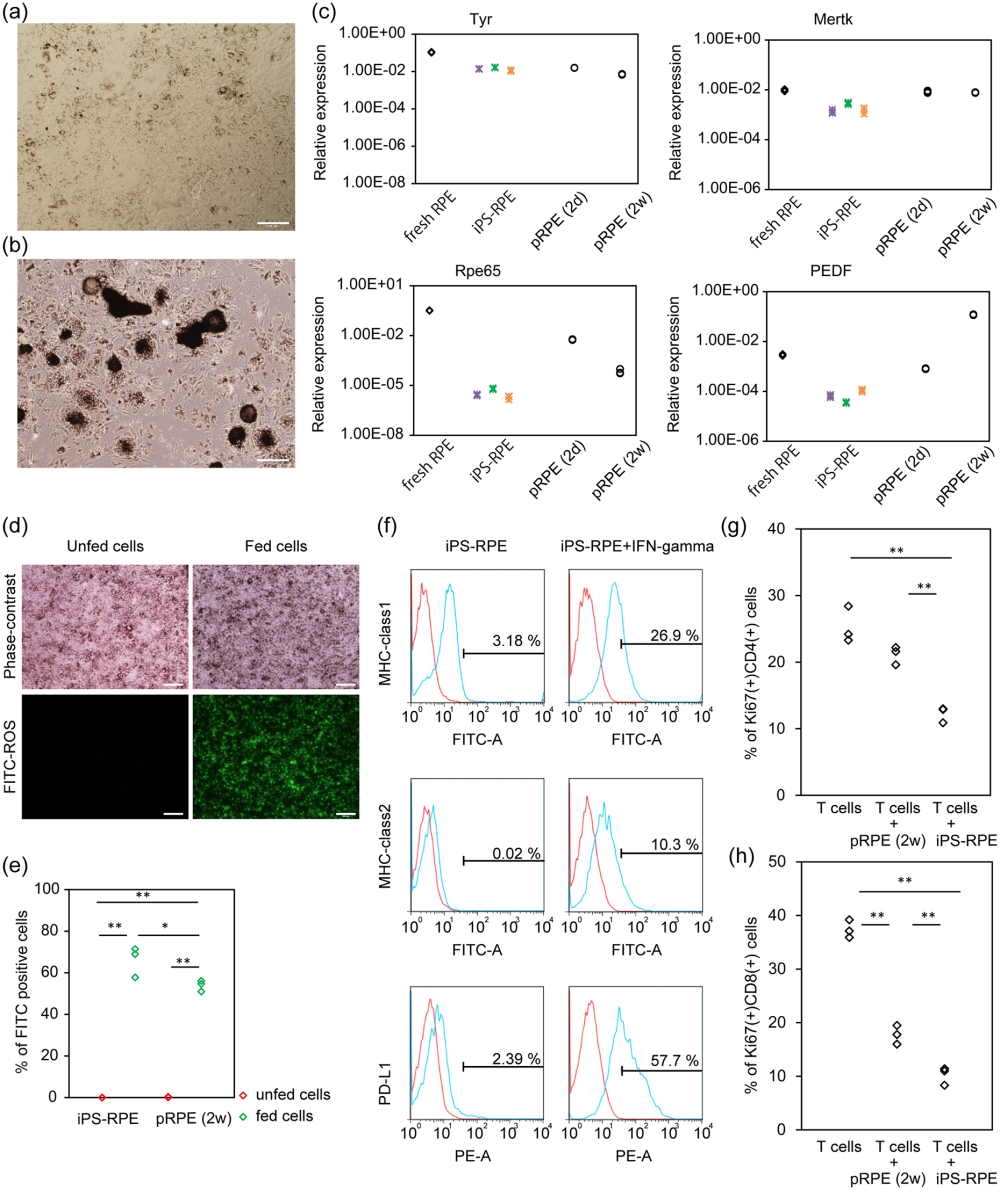
Functional characteristics of mouse iPS-RPE. (a and b) Two types of pRPE were compared with mouse iPS-RPE. Phase-contrast images of 2 week-cultured pRPE obtained from adult mice (pRPE (2w), a) and 2 day-cultured pRPE obtained from PN day10-mice (pRPE (2d), b) are shown. (c) The expression of RPE functional marker mRNAs in fresh PN day10-RPE, DD29 iPS-RPE, pRPE (2d), and pRPE (2w) is shown. The data for fresh RPE and iPS-RPE are the same as those shown in Fig 4. (d and e) Rod outer segment phagocytosis assay. Phase-contrast image and fluorescent image of iPS-RPE that was co-cultured with or without FITC-ROS and washed by medium are shown (d). Both iPS-RPE and pRPE (2w) phagocytosed FITC-ROS, and the percentage of FITC-positive cells in iPS-RPE was significantly higher than that in pRPE (2w). Tukey-Kramer test. n = 3. (f) Immune surface antigen expression evaluated by flow cytometry. The red line histograms represent isotype control. Numbers in the histogram indicate the percentage of positive cells. (g and h) Percentage of Ki-67 and CD4 double-positive T cells (g) and that of Ki-67 and CD8 double-positive T cells (h) after stimulation by anti-CD3 and anti-CD28 antibody. iPS-RPE significantly suppressed the proliferation of CD4-positive T cells and CD8-positive T cells. Tukey-Kramer test. n = 3. Scale bars: 200 μm (a, b) and 100 μm (d). **: p<0.01, *: p<0.05.

We used iPS-RPE between DD29 and DD39 for further characterization and functional studies, because expression levels of some functional genes (S3a Fig) and the morphology as evaluated by light microscopy did not significantly differ between these DDs. Since iPS-RPE may share some characteristics with primary cultures of mouse RPE cells (pRPE), which are widely used and more adequate for practical research use than freshly obtained cells, we first compared our iPS-RPE with two types of pRPEs: pRPE obtained from PN day10-mice and cultured for 2 days (these cells keep their cuboidal shape), and pRPE obtained from adult mice and cultured for 2 weeks (it is easy to obtain a large number of these cells, (Fig 5a and 5b)). The gene expression levels of Rpe65, Mertk and Tyr in cultured RPEs and iPS-RPE were lower than in freshly isolated RPE (Fig 5c). Among these three types of cultured RPEs, Rpe65, Mertk and Tyr expression levels were highest in the pRPE cultured for 2 days. PEDF expression was highest in the pRPE cultured for 2 weeks. The expression of phagocytosis-related gene Mertk was lower in iPS-RPE than in the two other types of cultured pRPE, but the difference seemed small.

Then, we evaluated the ability to phagocytose rod outer segment (ROS). FITC-ROS was loaded on iPS-RPE or 2 week-cultured mouse pRPE. The percentage of FITC-positive cells was analyzed by flow cytometry and compared with that of unfed RPEs. The percentage of FITC-positive cells was 66% ± 7.3% versus 0.03% ± 0.03% in iPS-RPE, and 53.9% ± 2.6% versus 0.29% ± 0.1% in pRPE (fed versus unfed RPE, mean ± S.D.). Both iPS-RPE and pRPE phagocytosed FITC-ROS, and the percentage of FITC-positive iPS-RPE cells was significantly higher than that of pRPE cells (p<0.05, Tukey-Kramer test, n = 3, Fig 5d and 5e), despite the slightly lower expression level of the phagocytosis-related gene Mertk.

From ICC evaluation, iPS-RPE expressed ZO-1 and P-cadherin (Fig 2a), which are both important factors for lateral cell contact between RPE. We evaluated the existence of functional tight junctions by transepithelial electrical resistance (TER). At DD29, TER in iPS-RPE cells was 59.0 Ωcm^2^ ± 31.7 Ωcm^2^ (mean ± S.D.).

Since one of our aims to use these cells is to study immune reactions after transplantation, we evaluated the expression patterns of cell surface antigens that are important for immune reactions (Fig 5f). Mouse iPS-RPE expressed MHC-class 1 under normal maintenance conditions. When iPS-RPE was stimulated by IFN-gamma, MHC-class 1 expression increased. MHC-class 2 and PD-L1 were also expressed by IFN-gamma treatment. We and others previously reported that RPE suppresses activated lymphocytes and suppresses pro-inflammatory reactions in intraocular spaces by using mouse pRPE and human iPS-RPE [1, 6, 7]. Thus, we evaluated whether our mouse iPS-RPE can also suppress lymphocyte proliferation by analyzing the positivity of Ki-67 staining by flow cytometry. When T cells were stimulated with anti-mouse CD3 antibody and anti-mouse CD28 antibody, T cells proliferated, and the percentage of Ki-67 and CD4 double-positive T cells was 25.3% ± 2.7%, and that of Ki-67 and CD8 double-positive T cells was 37.4% ± 1.7% (mean ± S.D.). When T cells were co-cultured with iPS-RPE, the percentage of Ki-67 and CD4 double-positive T cells was 12.3% ± 1.2%, and that of Ki-67 and CD8 double-positive T cells was 10.2% ± 1.7%. When T cells were co-cultured with 2 week-cultured mouse pRPE, the percentage of Ki-67 and CD4 double-positive T cells was 21.1% ± 1.4%, and that of Ki-67 and CD8 double-positive T cells was 17.8% ± 3.1% (mean ± S.D.). iPS-RPE significantly suppressed both CD4-positive T cells and CD8-positive T cells, and its suppression ability was stronger than that of mouse pRPE (p<0.01 for each, Tukey-Kramer test, Fig 5g and 5h and S3b Fig).

## Discussion

We successfully created a differentiation protocol to efficiently obtain retinal pigment epithelium (RPE) with high purity from mouse-induced pluripotent stem cells (iPSC), namely, about 2×10^6^ cells from 60 aggregates with approximately 98% purity. iPS-RPE exhibited a cobblestone appearance with cuboidal shape similar to typical RPE, and it expressed P-cadherin, which is expressed in RPE but not in retinal cells in mice [29, 30]. Our iPS-RPE also expressed ZO-1, which participates in adherence and tight junctions, in a polarized manner. The presence of tight junctions was also implied by elevated TER. Our group reported that TER values of several lines of human iPS-RPE ranged from 150 to 350 Ωcm^2^ [2]. Geisen et al. reported that TER of mouse primary RPE (pRPE) was 30 Ωcm^2^ [31]. In our study, the TER of mouse iPS-RPE was approximately 60 Ωcm^2^, and the TER of mouse iPS-RPE or pRPE would be lower than that of human iPS-RPE. In regard to supportive function for photoreceptor cells, the retinoid cycle and phagocytosis of rod outer segment (ROS) are important roles of RPE. Our mouse iPS-RPE phagocytosed rod outer segment comparable to pRPE, despite the lower gene expression of Mertk. In the retinoid cycle, 11-cis retinal is isomerized to all-trans retinal by photon absorption. Then, all-trans retinal is released from photoreceptor cells, and 11-cis retinal is regenerated by the reaction of several enzymes including Rpe65. Rpe65 expression is readily decreased in vitro, and it would be a sensitive marker to identify in vivo RPE [9, 32]. Rpe65 is expressed from the late embryonic period and increases to adult level in the postnatal period [33]. Expression, localization and function of Rpe65 are affected by transcription factors, soluble factors and cytoskeletal materials [34–37]. Sox9 is an important transcription factor of Rpe65 expression [34]. The expression level of Sox9 in iPS-RPE was slightly lower than that of freshly isolated RPE, and it would be one point to address when we want to improve our method. Although some soluble factors suppress Rpe65 expression [35, 36], as far as we know, there are no reports of soluble factors that accelerate Rpe65 expression. The additional knowledge about the cell maturation process is needed to improve the mouse iPS-RPE quality. Such knowledge would also be important for maintaining a stable quality of human iPS-RPE prepared from patients with various backgrounds, although there are some reports that human iPS-RPE expressed RPE65 at a similar level with that of human primary RPE [2] and that human iPS-RPE could contribute to the retinoid cycle [4, 38]. It is possible that the mouse iPS-RPE may express a higher level of Rpe65 in the in vivo environment after transplantation. Aoki et al. showed that pigmented cells differentiated from mouse embryonic stem cells (ESC) could express Rpe65 by further culture in chick embryo eye [18].

When we started adherent culture at DD7, we used laminin111. One reason for this choice was that previous investigators showed that laminin was expressed in the basal side of RPE at DD9 [19]. Another reason was that among several types of laminin synthesized by RPE [39], laminin111 is expressed from an early developmental stage [40, 41]. We continued to use laminin111 at DD11 because there was no obvious improvement in cell morphology with other coating materials we tried. DD11 cells could not form a sheet structure on Matrigel^®^ and collagen type 1, although these materials were used for human iPS-RPE differentiation and human fetal RPE culture [2, 17, 42, 43]. It would be another point to address to improve our protocol. Moreover, we examined if mouse iPS-RPE could be further passaged or stocked, but we had no success. We tried passage on DD14, DD15, DD23 and DD26 with Y27632 treatment, but the cells hardly proliferated. We tried to freeze cells on DD11 by using retinal differentiation medium 2 with CHIR99021, SU5402, Y27632, and 10% dimethyl sulfoxide. The thawed cells attached to laminin-coated dishes, but hardly proliferated. The difficulty in freezing and thawing these cells, and their low capacity for proliferation, would set some limitation for some volume experiments.

It is important to obtain substantial amount of cells in each differentiation in order to use iPS-RPE for research. Y27632 was useful for obtaining iPS-RPE efficiently. Y27632 inhibits Rho-associated, coiled-coil containing protein kinase, which contributes to stress fiber formation and actin filament stabilization [44, 45]. Y27632 also inhibits human iPSC/ESC apoptosis and supports the passage of human RPE derived from iPSC/ESC [42, 46]. When we used Y27632 during DD7 to DD11, not only the expansion of pigmented colonies, but also the adhesion of non-pigmented cells seemed to be improved. It is possible that Y27632 promoted colony expansion by influencing cytoskeleton formation, and it is also possible that some soluble factors from non-pigmented colonies promoted pigmented-colony expansion. The cell number we obtained at DD11 was not stable in each experiment and ranged from 9.68×10^5^ to 2.94×10^6^ cells. The cell number would be influenced by the differentiating state of DD7 aggregates, which are possibly influenced by the maintenance condition of iPSC. Previous investigators showed that the mouse strain influences the efficiency of creating ESC [47]. Hence, there are at least three points users of this protocol should keep in mind regarding its reproducibility: (1) mouse strain used, (2) maintenance condition of iPSC, and (3) differentiation condition of retinal progenitor cells. Although the cell number was not stable, the cell morphology evaluated by light microscopy and gene expression data were consistent in each experiment.

Lastly, as an immunologic feature, mouse iPS-RPE suppressed lymphocyte proliferation similar to human iPS-RPE and mouse pRPE in previous reports [1, 7]. The expression patterns of MHC-class 1 and MHC-class 2 were similar to those of previously reported human and monkey iPS-RPE [1, 2]. PD-L1 (B7H1) is a ligand of PD1, which is expressed on T cells. The interaction between PD-L1 and PD1 inhibits lymphocyte proliferation [48]. In human RPE, PD-L1 is constitutively expressed. In mouse pRPE, PD-L1 is not expressed constitutively, and its expression is induced by IFN-gamma treatment [49]. Likewise, our mouse iPS-RPE did not express PD-L1 under normal maintenance conditions, but expressed the molecule by IFN-gamma treatment, which is a response that is comparable to mouse pRPE [49]. These results suggest that mouse iPS-RPE may behave differently from human RPEs, but may still be able to suppress activated T cells via cell-to-cell contact because activated T cells produce inflammatory cytokines such as IFN-gamma that may induce PD-L1 on mouse iPS-RPE. It was reported that mouse pRPE suppresses immune cells also by soluble factors such as transforming growth factor, which is similar to the process in human iPS-RPE [1, 50]. These immunologic properties of mouse iPS-RPE will allow us to study immunologic reactions after human iPS-RPE transplantation, including rejection and graft integration.

## Conclusion

In the present study, we describe the protocol for differentiation of mouse iPS-RPE with high purity. Mouse iPS-RPE showed typical cell morphology and characteristic function similar to human iPS-RPE, including immunological function. Mouse iPS-RPE can serve as an attractive tool for functional and morphological studies of RPE.

## Materials and Methods

All animal experiments were conducted with the approval of the RIKEN Center for Developmental Biology Ethical Committee (No. AH18-05-23). All animal samples were obtained after rapid sacrifice by cervical dislocation, and all efforts were made to minimize suffering.

### Maintenance of mouse induced pluripotent stem cells (iPSC)

We used Nrl-GFP iPSC that were generated [51] from Nrl-GFP transgenic mice [52]. Nrl-GFP iPSC was maintained in GMEM (11710–035, Thermo Fisher Scientific) / 10% FBS (555–21245, Biosera)/1 mM sodium pyruvate (S8636, Sigma) / 0.1 mM NEAA (11140–050, Thermo Fisher Scientific) / 0.1 mM 2-mercaptoethanol (2-ME, 137–06862, Wako) supplemented with 1000 U/ml LIF (ESG1107, Esgro), 3 μM CHIR99021 (1677–25, BioVision), and 1 μM PD0325901 (04-0006-10, Stemgent) on 0.1% gelatin-coated dish (G2625, Sigma). Cells were dissociated by 3–4 days using 0.25% trypsin-1 mM EDTA (25200, Thermo Fisher Scientific), and 0.75–1.5 x 10^5^ cells were seeded in a 60-mm dish.

### Differentiation of optic-vesicle structure

3D optic-vesicle structures were differentiated by the retinal serum-free floating culture of embryoid body-like aggregates with quick reaggregation (SFEBq) method [19] with minor modifications [53, 54]. In brief, embryoid bodies were formed by putting 3000 cells/well of Nrl-GFP iPSC in low-cell binding 96-well plates (174925, Nunc) in modified retinal differentiation medium (GMEM / 5% KSR (10828–028, Thermo Fisher Scientific) / 0.1 mM NEAA / 1 mM sodium pyruvate / 0.1 mM 2-ME) supplemented with 0.1 μM AGN193109 (A427000, Toronto Research Chemicals). At differentiation day (DD) 1, Matrigel^®^ (354230, Corning) was added to a final concentration of 2%. At DD7, a portion of aggregates was transferred to floating culture with retinal maturation medium 1 (DMEM/F12 with GlutaMAX (10565, Gibco) / 1% N2 supplement (17502–048, Thermo Fisher Scientific) / 100 PU/ml penicillin and streptomycin (15140–122, Thermo Fisher Scientific)) to confirm retinal differentiation. Most of the aggregates were used for RPE differentiation.

### Adherence and induction to RPE

At DD7, aggregates were picked up into a 60-mm dish with retinal maturation medium 2 (retinal maturation medium 1 with 10% FBS) and were broken up by passing first through 23-gauge and then 25-gauge needles with a 1-ml sterile plastic syringe. Cell fragments and debris were removed by centrifugation (500 rpm, 2 min with slow acceleration and slow deceleration) two times and filtration by using a cell strainer (352235, Corning). The remaining cell clumps and sheets were placed on the laminin (1 μl/cm^2^, L2020, Sigma) -coated dish in retinal maturation medium 2 supplemented with 3 μM CHIR99021, 5 μM SU5402 (SML0443, Sigma) and 10 μM Y27632 (253–00513, Wako). Thirty to forty aggregates were used for one 60-mm dish.

### Purification of adhesive pigmented cells

At DD11, expanded colonies were treated with Accutase^®^ (SCR-005, Millipore) for 5 min at 37°C. After adding retinal maturation medium 2 for neutralization, most non-pigmented colonies and less adhesive pigmented cells were removed by intense pipetting (about 20 to 30 times). A small amount of adhesive non-pigmented cells was removed manually. The remaining adhesive pigmented cells were treated by 3 min of 37°C incubation with 0.25% trypsin-1 mM EDTA. Retinal maturation medium 2 was added for neutralization of trypsin, and cells were scraped with a cell scraper (MS-93100, Sumitomo Bakelite) and were completely dissociated by pipetting. Dissociated cells were put on a laminin-coated well. Cell number and laminin concentration are shown in S1 Table. Other coating materials that we tried were fibronectin (5 μg/ml, 50 μg/ml, 33016, Thermo Fisher Scientific), collagen type 4 (5 μg/cm^2^, 10 μg/cm^2^, C5533, Sigma), 0.1% gelatin, collagen type 1 (Cellmatrix Type 1A^®^, Nitta Gelatin), Extracellular Matrix (7 μg/cm^2^, 354237, Corning), CELLstart^®^ (20 ul/ml, A1014201, Thermo Fisher Scientific), and Matrigel^®^ (2.5%, 10%, 40%, and 100%).

### Maturation of RPE

At DD12, the same volume of retinal maturation medium 2 supplemented with CHIR99021 and SU5402 was added to dilute Y27632. If cells did not completely cover the surface of the culture well, this procedure was skipped. At DD13, medium was changed to retinal maturation medium 2 supplemented with CHIR99021 and SU5402 without Y27632. At DD16, medium was changed to MEM / N1 / FBS medium (MEM-alpha (M-4526, Sigma) / 1% FBS / 1% N1 supplement (N-6530, Sigma) / 2 mM L-Glutamine (G7513, Sigma) / 0.1 mM NEAA / 250 mg/L L-taurine (T8691, Sigma) / 20 μg/L hydrocortisone (H-0396, Sigma) / 0.013 μg/L triiodo-thyronine (T-5516, Sigma)) which was used for human fetal RPE culture [43]. The medium was changed three times per week.

### Confirmation of retinal progenitor cells

At DD7, three to six aggregates were differentiated into retinal structure as described previously with minor modifications [53, 55]. In brief, aggregates were transferred to floating culture with retinal maturation medium 1 and cultured in a 37°C, 40% O_2_, 5% CO_2_ atmosphere. Medium was changed in 2–3 days. From DD14, medium was supplemented with 0.5 μM retinoic acid (R2625, Sigma) and 1 mM L-taurine. At DD26, expression of Nrl-GFP was confirmed by fluorescent microscopy (X71, Olympus).

### Control RPE samples

As control RPE for marker gene expression, we freshly isolated RPE from postnatal (PN) day10 C57BL/6 (B6) mice as previous described [56]. We also used PN day10-RPE as a control sample for immunocytochemistry by immediate fixation just after enucleation. To compare the cell characteristics and functions of iPS-RPE with those of primary RPE (pRPE), we used two types of pRPE: 2-day cultured pRPE obtained from PN day10-B6 mice and 2-week cultured pRPE obtained from 6-week-old B6 mice. Detailed protocols are provided in the S1 Text.

### Immunocytochemistry and imaging

For immunocytochemistry, dissociated pigment cells were put on laminin-coated 8-well slide glass (S1 Table) at DD11. Samples were fixed for 15 min with 4% paraformaldehyde / phosphate buffered saline (PBS) at room temperature or for 30 min in -30°C methanol. Fixed samples were incubated for 30 min in 0.3% Triton X-100 (T9284, Sigma) / PBS for permeation, and for 1 h in 5% goat serum / 0.3% Triton X-100 / PBS for blocking at room temperature. First antibodies were diluted by 1% goat serum / 0.3% Triton X-100 / PBS, and samples were incubated overnight at 4°C. Dilution and fixation conditions for each antibody are listed in S2 Table. Secondary antibody conjugated with Alexa Fluor 546 or Alexa Fluor 647 was incubated for 1 h at room temperature. The samples were mounted in FluorSave Reagent (345789, Merck Millipore). Tile scan images of F-actin (Phalloidin) were obtained with a Leica TCS SP8 (Leica) confocal laser microscope. Other confocal images were obtained from LSM700 (Zeiss).

### Electron microscopy

Samples were fixed with 2% fresh formaldehyde and 2.5% glutaraldehyde in 0.1 M sodium cacodylate buffer (pH 7.4) for 2 h at room temperature. After washing with 0.1 M cacodylate buffer (pH 7.4) three times, they were postfixed with ice-cold 1% OsO4 in the same buffer for 2 h. The samples were rinsed with distilled water, stained with 0.5% aqueous uranyl acetate for 2 h or overnight at room temperature, dehydrated with ethanol and propylene oxide, and embedded in Poly/Bed 812 (Polyscience). Ultra-thin sections were cut, doubly-stained with uranyl acetate and Reynold’s lead citrate, and viewed with a JEM 1400 plus transmission electron microscope (JEOL) at an accelerating voltage of 100 kV.

### Effect of Y27632 on cell number

To evaluate the effect of Y27632 on cell proliferation, aggregates were broken up and put on three laminin-coated 35-mm dishes on DD7 in each differentiation, with or without Y27632. Thirty aggregates without Y27632 and 20 aggregates with Y27632 were plated on one dish because both pigmented cell and non-pigmented cell attachments seemed to be promoted by Y27632 treatment. Three dishes were separately counted for adhesive pigment cell number at DD11. The mean cell number of three dishes without Y27632 (30 aggregates/dish) was doubled, and the mean cell number of three dishes with Y27632 (20 aggregates/dish) was tripled for comparison as of subsequent cell number per 60 aggregates (n = 7 experiments). Dissociated cells from each dish were passaged separately into each well of a 96-well plate, and individual wells were evaluated for purity as described below.

### Purity of iPS-RPE

At DD29, mouse iPS-RPE was fixed by a 30-min treatment in -30°C methanol. PBS was loaded in each well, and photos were taken by using a motorized inverted system microscope (IX71, Olympus), charge-coupled device camera (DP72, Olympus), and digital imaging software (DP2-BSW, Olympus). The bright-field RGB images (2070 × 1548 pixels) were captured under the same conditions (dark room, objective lens: Olympus UPlanFL 10 × N.A. 0.30, exposure time: 0.5 ms, and ISO: 400). Image analysis to evaluate the percentage of pigmented cell area was performed with ImageJ analysis software by thresholding, segmentation by watershed algorithm, and using the ‘analyze particles’ tool to obtain the number of pixels representing the area of less pigmented cells. The proportion of pigmented cells to total area of the images was expressed as a percent.

### Pigment intensity evaluation by flow cytometry

Mouse iPS-RPE was dissociated by a 5-min treatment with 0.25% trypsin-1 mM EDTA and fixed by a 15-min treatment with Cytofix / Cytoperm Kit (554714, BD). DD30—DD36 iPS-RPE was used for this assay. Cells were gated and separated into 2 populations on the basis of side-scattering (SSC) properties by using FACSAria^®^ II (BD). To evaluate whether the SSC property reflected the intensity of pigmentation, 5000 cells/well of each population were put in a low-cell binding 96-well plate. Bright field photos were obtained by microscope, as stated above, using a UPlanFL 4 × N.A. 0.30 objective lens and 0.2-ms exposure time. The mean intensity of all image areas was analyzed using Image J analysis software, and the significance of the difference was evaluated between the SSC-high population and SSC-low population.

### Real-time quantitative reverse transcription polymerase chain reaction (qRT-PCR)

For PCR analysis, cells were collected by adding RNAprotect^®^ reagent (76526, Qiagen) directly into RPE culture wells. RNA was extracted with an RNeasy micro kit (74004, Qiagen). A total of 500 ng to 1 μg RNA was used for making 20 μl of cDNA by using a Transcriptor First Strand cDNA kit (04897030001, Roche). The cDNA was amplified with gene-specific primers and probes designed using the Roche Universal Probe Library (S3 Table) and LightCycler^®^ 480 Probes Master (04707494001, Roche). qRT-PCR was performed by using LightCycler^®^ 480 (Roche) and samples were run in triplicate, and at least three independent experiment cultures were analyzed. The relative expression level of each gene was normalized by Gapdh.

### Transepithelial electrical resistance (TER)

TER was performed at DD29 as previously described [2] by Millicell^®^ ERS-2 (Millipore). Net TER (Ωcm^2^) was calculated by subtracting the value of laminin-coated insert as a blank from the experimental value and multiplying the area of the insert membrane.

### Rod outer segments (ROS) phagocytosis assay

ROS was isolated from fresh porcine retinas as previously described [57], and was labeled with FITC dye (F1906, Thermo Fisher Scientific) following the manufacturer's recommended protocol. Briefly, 10 mg/ml ROS in 0.1 M sodium bicarbonate buffer (pH 9.0) was incubated in 1 mg/ml FITC for 1 h at room temperature in the dark and was then washed and resuspended in 5% sucrose at 1 mg/ml. FITC-ROS was resuspended in MEM / N1 / FBS medium at a concentration of 10 μg/cm^2^. FITC-ROS was loaded in the DD37 iPS-RPE culture well or 2 week-cultured pRPE. Cells were further incubated in a 37°C, 5% CO_2_ atmosphere for 24 h. iPS-RPE and pRPE were dissociated by a 5-min treatment with 0.25% trypsin-1 mM EDTA and were analyzed for the FITC-positive ratio by flow cytometry (FACSCanto^®^ 2, BD).

### Evaluation of immunological antigen expression

Expression levels of immunological antigens were evaluated by flow cytometry (FACSCanto^®^ 2, BD). FITC-conjugated anti-MHC-class 1 mAb (MCA2189F, AbD Serotec), FITC-conjugated anti-MHC-class 2 mAb (11–5321, eBioscience), and PE-conjugated anti-PD-L1 mAb (124307, BioLegend) were used to stain iPS-RPE incubated with or without 72 h of 100 ng/ml IFN-gamma (554587, BD Pharmingen^®^). Before staining, the iPS-RPE was incubated with mouse Fc block (553141, BD Pharmingen^®^) for 15 min at 4°C. As isotype controls, we used FITC-conjugated mouse IgG isotype (555057, BD Pharmingen^®^), FITC-conjugated rat IgG isotype (11-4031-81, eBioscience), and PE-conjugated rat IgG isotype (400608, BioLegend).

### Lymphocyte proliferation assay

Spleens were removed from allogeneic BALB/c mice and were pressed through a 100-μm cell strainer to produce a single-cell suspension of cells. Red blood cells were dissolved using ACK lysing buffer (10-548E, Lonza). Pan-T cells were purified using a cell isolation kit (MACS system: 130-095-130, Miltenyi Biotec). For anti-CD3 and anti-CD28-driven T cell activation, 1 × 10^6^ cells/well of purified T cells were added to the 1 × 10^5^ cells/well of iPS-RPE or 2-week cultured pRPE with 200 ul of lymphocyte medium (RPMI-1640 (30264, Nacarai) / 10% FBS / 100 PU/ml penicillin and streptomycin). RPE and T cells were co-cultured at 37°C in a 5% CO_2_ atmosphere for 72 h. After a 72-h incubation, the T cells were evaluated by staining with FITC-conjugated anti-Ki-67 mAb (652410, BioLegend), co-stained with PE-conjugated anti-CD4 mAb (553730, BD Pharmingen^®^) or APC-conjugated anti-CD8 mAb (100711, BioLegend). As an isotype control, we used FITC-conjugated rat IgG isotype (400505, BioLegend), PE-conjugated rat IgG isotype (553989, BD Pharmingen^®^), and APC-conjugated rat IgG isotype (100711, BioLegend).

### Statistical methods

Appropriate statistical tests were applied, including the Mann-Whitney U test, chi-square test, and Tukey-Kramer test. All statistical analyses were conducted using Statcel statistical package (Statcel3; OMS Inc.). *P* values <0.05 were considered to be statistically significant.

## Supporting Information

S1 Fig Supporting information for the procedure. (a, b) Merged images of DAPI staining (blue) and bright field images of before (a) and after (b) Accutase^®^ treatment. Most non-pigmented colonies (DAPI-positive, pigment-negative) were removed by Accutase^®^ treatment. Dotted circle lines in (b) indicate remaining adhesive non-pigmented cells that were removed manually. (c and d) Effect of cell dissociation and Y27632 supplement on cell attachment at DD7 (c) and DD11 (d). Phase-contrast images at DD11 with or without dissociation on DD7 and/or Y27632 supplement (c) and images that were taken 1 day after passage on DD11 with or without Y27632 supplement (d) are shown. (e) Phase-contrast images of DD29 cells passaged on collagen type 1 and various concentrations of Matrigel^®^ are shown. Cells formed clusters on 40% and 100% Matrigel^®^. (f) Phase-contrast image of patchy region seen in DD29 cells cultured on glass (inside the dotted line). Such regions lost the ZO-1 expression evaluated by immunocytochemistry (g). (h) The patchy regions were seen when cells were cultured on glass. chi-square test. n = 28 for ‘on glass’ group, n = 58 for ‘on polystyrene’ group, both from five independent experiments. Scale bars: 500 μm (a-c, e and f) and 100 μm (d and g). **: p<0.01.
(TIF)

S2 Fig Supporting information for morphological evaluation. (a) Immunocytochemistry of F-actin (Phalloidin-Alexa 546) and Sox9. Nuclei were counter-stained with DAPI. Nuclear staining patterns of Sox9 were confirmed both in DD29 cells maintained with MEM / N1 / FBS medium and PN day10-mouse RPE. (b and c) Electron microscopy image of DD29 cells without passage at DD11. Magnified view of the area in the yellow square is shown in (c) to show long, fine microvilli. (d) Phase-contrast image of the DD29 cells without passage at DD11. Magnified image (e) shows that cells in the peripheral region did not become pigmented even though these cells formed a cuboidal shape (arrow head). (f) Cells in the most peripheral region could not form a cuboidal shape as evaluated by F-actin staining (arrow). A magnified view of the yellow square area indicates that cells in the middle of the colony could form a cuboidal shape like the cells that were passaged on DD11. Scale bars: 500 μm (d, f), 100 μm (a, e), 10 μm (b) and 2 μm (c).
(TIF)

S3 Fig Support information for characteristics of iPS-RPE. (a) Transcriptional analyses of iPS-RPE on DD29 and DD36. Values were normalized by Gapdh. Each marker is presented as a mean value of triplicate evaluation. Data from 4 independent experiments are shown in different colors. (b) Representative data from three experiments of the lymphocyte proliferation assay. T cells without co-culture and T cells co-cultured with iPS-RPE or pRPE (2w) were stained with anti-Ki-67 antibody and anti-CD4 antibody or anti-CD8 antibody. Values on the histograms indicate the percentage of cells double-positive for Ki-67 and CD4 or Ki-67 and CD8.
(TIF)

S1 Table Cell number and laminin111 concentration at DD11 passage. PBS; phosphate buffered saline.
(DOCX)

S2 Table Antibody and fixation condition for immunocytochemistry. (DOCX)

S3 Table Probe and primer design for real-time quantitative reverse transcription polymerase chain reaction. (DOCX)

S1 Text Protocol for obtaining control RPE sample from eyes of C57BL/6 mice. (DOCX)
