# Supplementary figures and images for: Differentiation/Purification Protocol for Retinal Pigment Epithelium from Mouse Induced Pluripotent Stem Cells as a Research Tool
Source: PLoS One. 2016 Jul 6;11(7):e0158282. doi: 10.1371/journal.pone.0158282 (PMC4934919; doi:10.1371/journal.pone.0158282)

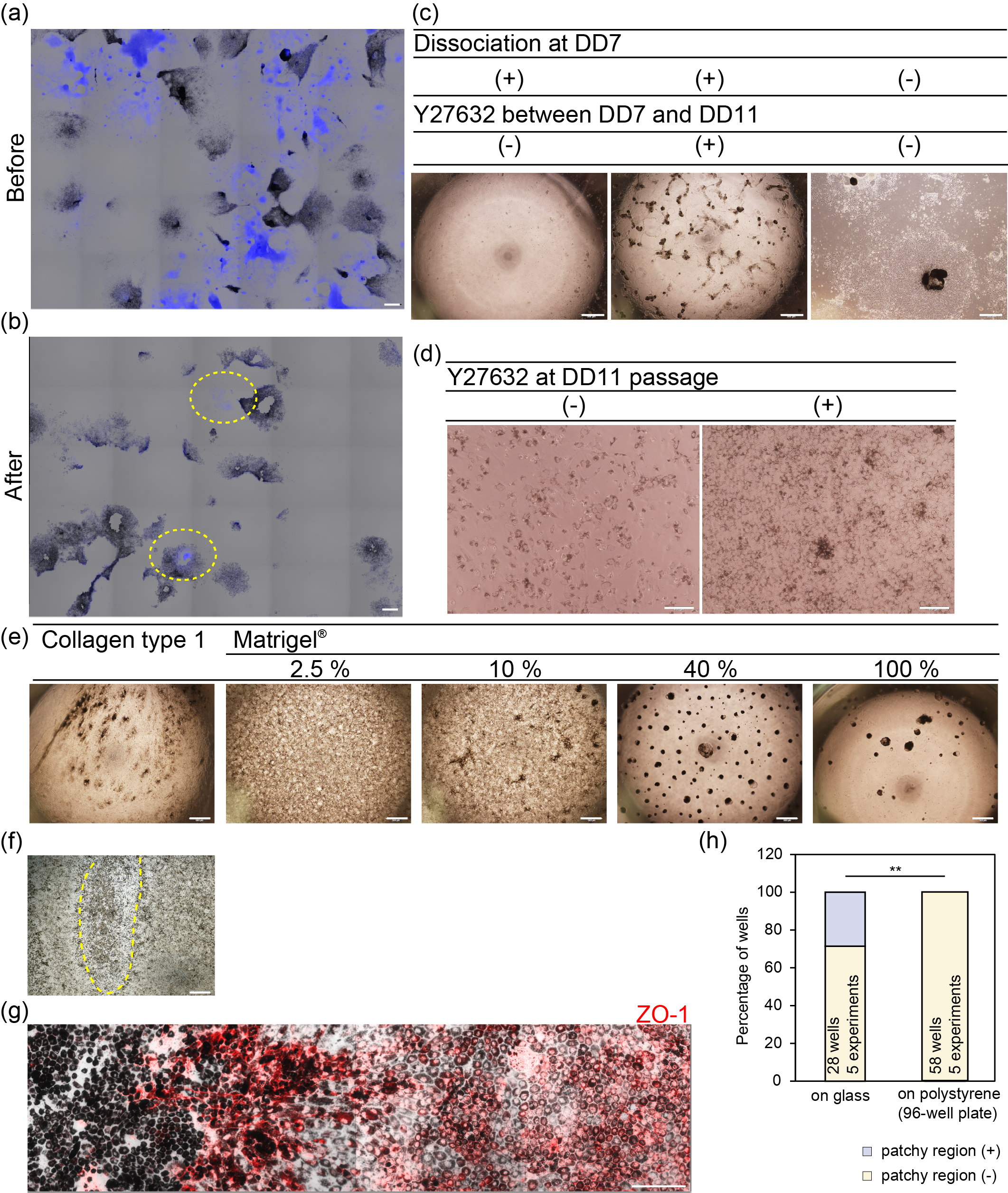

Supplement: S1 Fig — (a, b) Merged images of DAPI staining (blue) and bright field images of before (a) and after (b) Accutase® treatment. Most non-pigmented colonies (DAPI-positive, pigment-negative) were removed by Accutase® treatment. Dotted circle lines in (b) indicate remaining adhesive non-pigmented cells that were removed manually. (c and d) Effect of cell dissociation and Y27632 supplement on cell attachment at DD7 (c) and DD11 (d). Phase-contrast images at DD11 with or without dissociation on DD7 and/or Y27632 supplement (c) and images that were taken 1 day after passage on DD11 with or without Y27632 supplement (d) are shown. (e) Phase-contrast images of DD29 cells passaged on collagen type 1 and various concentrations of Matrigel® are shown. Cells formed clusters on 40% and 100% Matrigel®. (f) Phase-contrast image of patchy region seen in DD29 cells cultured on glass (inside the dotted line). Such regions lost the ZO-1 expression evaluated by immunocytochemistry (g). (h) The patchy regions were seen when cells were cultured on glass. chi-square test. n = 28 for ‘on glass’ group, n = 58 for ‘on polystyrene’ group, both from five independent experiments. Scale bars: 500 μm (a-c, e and f) and 100 μm (d and g). **: p<0.01. (TIF) [file pone.0158282.s001.tif]

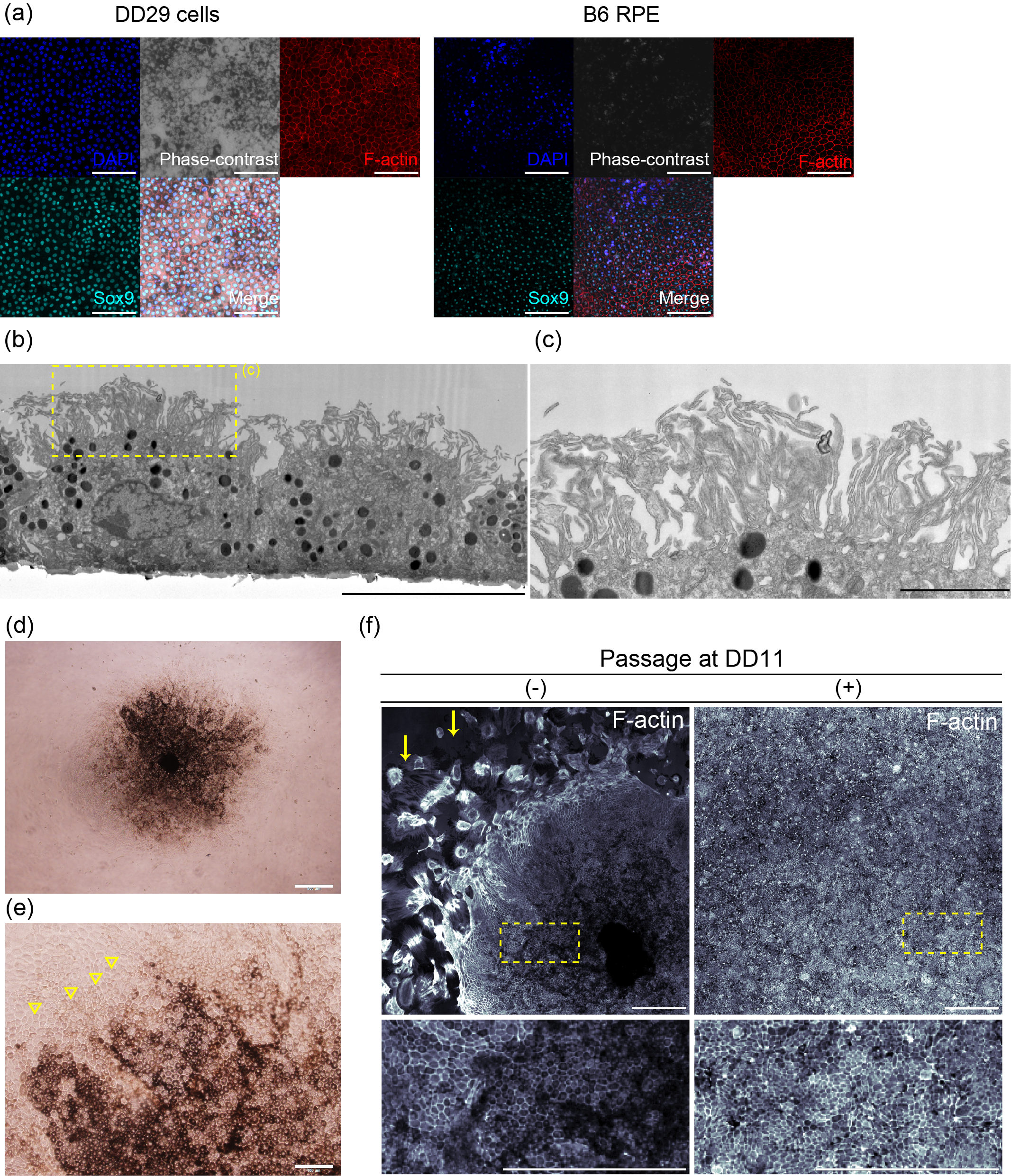

Supplement: S2 Fig — (a) Immunocytochemistry of F-actin (Phalloidin-Alexa 546) and Sox9. Nuclei were counter-stained with DAPI. Nuclear staining patterns of Sox9 were confirmed both in DD29 cells maintained with MEM / N1 / FBS medium and PN day10-mouse RPE. (b and c) Electron microscopy image of DD29 cells without passage at DD11. Magnified view of the area in the yellow square is shown in (c) to show long, fine microvilli. (d) Phase-contrast image of the DD29 cells without passage at DD11. Magnified image (e) shows that cells in the peripheral region did not become pigmented even though these cells formed a cuboidal shape (arrow head). (f) Cells in the most peripheral region could not form a cuboidal shape as evaluated by F-actin staining (arrow). A magnified view of the yellow square area indicates that cells in the middle of the colony could form a cuboidal shape like the cells that were passaged on DD11. Scale bars: 500 μm (d, f), 100 μm (a, e), 10 μm (b) and 2 μm (c). (TIF) [file pone.0158282.s002.tif]

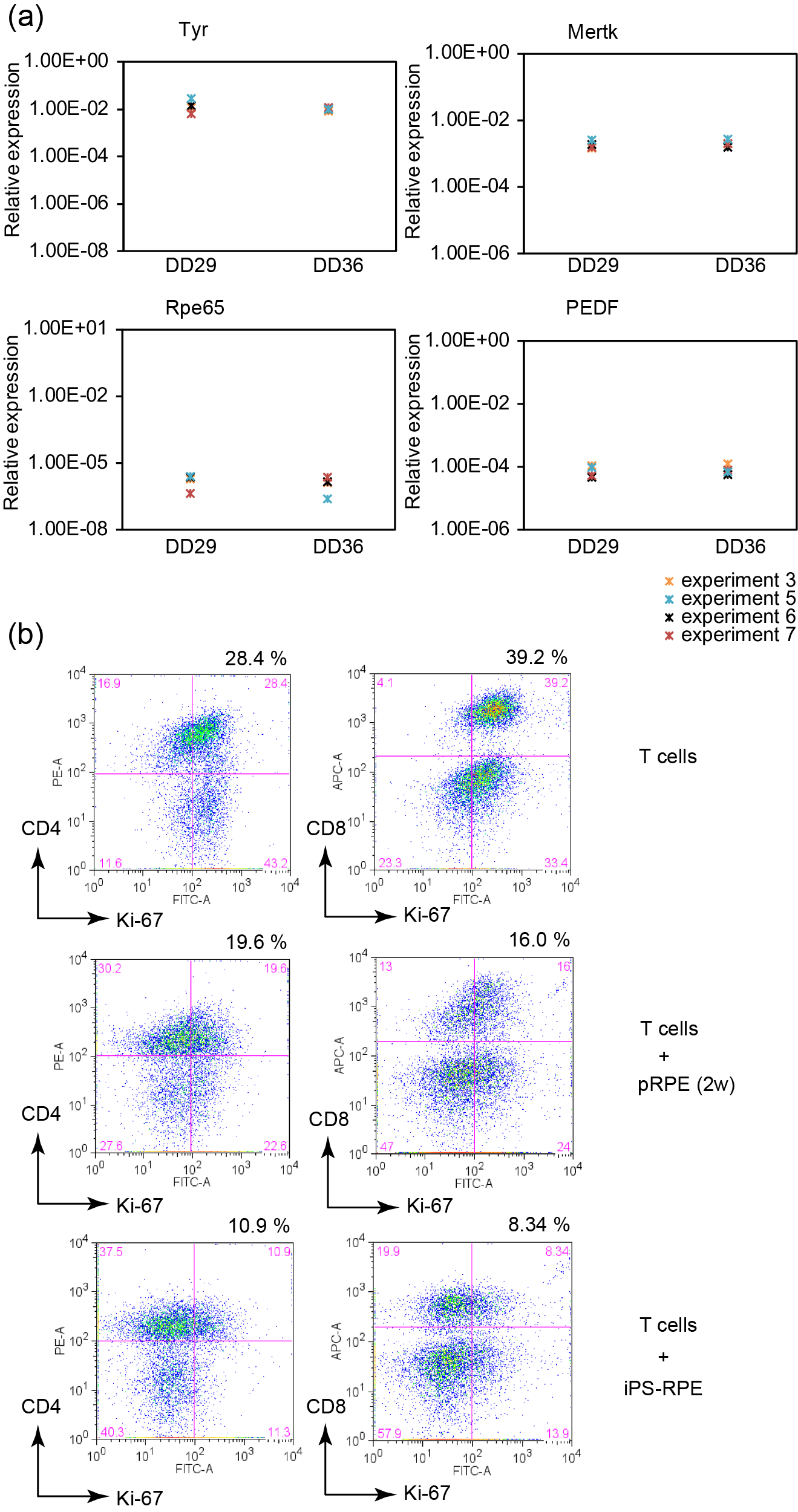

Supplement: S3 Fig — (a) Transcriptional analyses of iPS-RPE on DD29 and DD36. Values were normalized by Gapdh. Each marker is presented as a mean value of triplicate evaluation. Data from 4 independent experiments are shown in different colors. (b) Representative data from three experiments of the lymphocyte proliferation assay. T cells without co-culture and T cells co-cultured with iPS-RPE or pRPE (2w) were stained with anti-Ki-67 antibody and anti-CD4 antibody or anti-CD8 antibody. Values on the histograms indicate the percentage of cells double-positive for Ki-67 and CD4 or Ki-67 and CD8. (TIF) [file pone.0158282.s003.tif]
