# Supplementary material for: Differentiation/Purification Protocol for Retinal Pigment Epithelium from Mouse Induced Pluripotent Stem Cells as a Research Tool
Source: PLoS One. 2016 Jul 6;11(7):e0158282. doi: 10.1371/journal.pone.0158282 (PMC4934919; doi:10.1371/journal.pone.0158282)
Supplement: S1 Table — PBS; phosphate buffered saline. (DOCX) [file pone.0158282.s004.docx]

**S1 Table: Cell number and laminin111 concentration at DD11 passage.**

|  | **Cat number, company** | **Dish material** | **Surface treatment** | **Cell growth area** | **Laminin111 coating per well** | **Cell number per well** |
| --- | --- | --- | --- | --- | --- | --- |
| **96-well plate** | 353072, BD | Polystyrene | Tissue culture treated | 0.32 cm^2^ | 0.33 µl / 70 µl PBS | 1 x 10^5^ cells |
| **24-well plate** | 353047, BD | Polystyrene | Tissue culture treated | 2.0 cm^2^ | 2.0 µl / 500 µl PBS | 5.5 x 10^5^ cells |
| **8-well slide glass** | 354632, BD | Glass | Poly-D-lysine- coated | 0.7 cm^2^ | 1.4 µl / 300 µl PBS | 2 x 10^5^ cells |
| **Transwell** | 3470, Corning | Polyethylene | Tissue culture treated | 0.33 cm^2^ | 0.66 µl / 70 µl PBS | 1 x 10^5^ cells |
