# Supplementary material for: Differentiation/Purification Protocol for Retinal Pigment Epithelium from Mouse Induced Pluripotent Stem Cells as a Research Tool
Source: PLoS One. 2016 Jul 6;11(7):e0158282. doi: 10.1371/journal.pone.0158282 (PMC4934919; doi:10.1371/journal.pone.0158282)
Supplement: S2 Table — (DOCX) [file pone.0158282.s005.docx]

**S2 Table: Antibody and fixation conditions for immunocytochemistry.**

| **Antigen** | **Host** | **Fixation conditions** | **Dilution** | **Cat number, company** |
| --- | --- | --- | --- | --- |
| **ZO-1** | Rb | Methanol, -30℃, 30 min | × 250 | 61-7300, Thermo Fisher Scientific |
| **P-cadherin** | Rt | 4% paraformaldehyde, 4℃, 15 min | × 250 | MAB761, R&D Systems |
| **Sox9** | Rb | 4% paraformaldehyde, 4℃, 15 min | × 500 | AB5535, EMD Millipore |
| **Phalloidin-Alexa 546** | (-) | 4% paraformaldehyde, 4℃, 15 min | × 100 | A22283, Thermo Fisher Scientific |
