# Supplementary material for: Differentiation/Purification Protocol for Retinal Pigment Epithelium from Mouse Induced Pluripotent Stem Cells as a Research Tool
Source: PLoS One. 2016 Jul 6;11(7):e0158282. doi: 10.1371/journal.pone.0158282 (PMC4934919; doi:10.1371/journal.pone.0158282)
Supplement: S3 Table — (DOCX) [file pone.0158282.s006.docx]

**S3 Table: Probe and primer design for real-time quantitative reverse transcription polymerase chain reaction.**

| **Gene-Specific Primers** | | | | |
| --- | --- | --- | --- | --- |
| **Gene** | **Left primer** | **Right primer** | **Amplicon size (nt)** | **Probe number** |
| **Gapdh** | agcttgtcatcaacgggaag | tttgatgttagtggggtctcg | 62 | #9 |
| **Rx** | cgacgttcaccacttaccaa | tcggttctggaaccatacct | 140 | #78 |
| **Mitf** | tgaagcaagagcattggcta | gttaaatcttcttcttcgttcaatca | 76 | #34 |
| **Mertk** | gttctggccccactgcta | aaaggccctgaaaatagctga | 110 | #66 |
| **Tyr** | tcgtcaccctgaaaatcctaa | ctgatctgctacaaatgatctgc | 77 | #26 |
| **Serpinf1 (PEDF)** | ggactctgatctcaactgcaag | aagttctgggtcacggtcag | 93 | #4 |
| **Rpe65** | tcaggagatatgtacttcctttgaca | ttgtatggggcagtgtgact | 75 | #96 |
| **Pax6** | gcttggtggtgtctttgtca | tgcatctgcatgggtctg | 127 | #29 |
| **Sox9** | gtacccgcatctgcacaac | ctcctccacgaagggtctct | 94 | #66 |
| **Otx2** | aaatcaacttgccagaatcca | ggcctcactttgttctgacc | 109 | #84 |
