# Supplementary material for: Differentiation/Purification Protocol for Retinal Pigment Epithelium from Mouse Induced Pluripotent Stem Cells as a Research Tool
Source: PLoS One. 2016 Jul 6;11(7):e0158282. doi: 10.1371/journal.pone.0158282 (PMC4934919; doi:10.1371/journal.pone.0158282)
Supplement: S1 Text — (DOCX) [file pone.0158282.s007.docx]

**S Text: Protocol for obtaining control RPE samples from eyes of C57BL/6 mice**

**Freshly isolated RPE for RNA control samples**

RPE was isolated as previously reported [56] with minor modifications. After cervical dislocation, the eyes were enucleated and circumferential cuts were made just posterior to the ciliary processes. The lens, vitreous and sensory retina were removed. The remaining posterior eye cups were placed into RNAprotect^®^ reagent (76526, Qiagen) for 10 to 30 min at room temperature. Then, the posterior eye cups were removed from the solution, and RPE cells were collected by centrifugation (3200 rpm, 10 min). From RPE obtained from 14 eyes enucleated from seven postnatal (PN) day 10-mice, a total of approximately 3 μg of RNA was extracted.

**Two day-cultured primary RPE (pRPE)**

Two day-cultured pRPE was obtained as previously reported [11, 43]. In brief, 20 eyes were enucleated from ten PN day 10-mice and washed first with PBS and second with growth medium 1 (DMEM with high glucose (D5796, Sigma) / 0.1 mM NEAA / 100 PU/ml penicillin and streptomycin). Eyes were incubated with 2% Dispase^®^ (04942078001, Roche) in growth medium 1 for 45 min at 37℃. Eyes were then washed with growth medium 2 (growth medium 1 / 10% FBS). Posterior eye cups were made as described above, and RPE were peeled as sheets by fine forceps. RPE sheets were collected in 1.5 ml tubes, washed twice with growth medium 2, and dissociated by 0.05% trypsin treatment (15090-046, Thermo Fisher Scientific) for less than 5 min. Cells were washed and resuspended in growth medium 2 and then seeded in two wells of a 12-well plate and incubated at 37℃ in a 5% CO_2_ atmosphere for 2 days.

**Two week-cultured pRPE**

Two week-cultured pRPE were obtained as described previously [6] with minor modifications. Two eyes were enucleated from one 6-week-old B6 mouse. Posterior eye cups were incubated in 0.25% trypsin-1mM EDTA for 1 h at 37℃ in a 5% CO_2_ atmosphere. RPE cells were peeled off gently as sheets with fine forceps. RPE sheets were progressively triturated, first through a 21-gauge needle and then a 23-gauge needle, to create a single-cell suspension. After washing twice and resuspending in growth medium (DMEM/F12 (D6421, Sigma) / 10% FBS / 100 PU/ml penicillin and streptomycin), RPE cells were seeded in one well of a 6-well plate and incubated at 37℃ in a 5% CO_2_ incubator for 2 weeks.
